# Therapeutic Challenges with Catastrophic Antiphospholipid Antibody Syndrome: A Case Report

**DOI:** 10.7759/cureus.10482

**Published:** 2020-09-16

**Authors:** Prateek Harne, Parth J Sampat, Maneesh Bisen, Jihad BenGabr, Hom Neupane

**Affiliations:** 1 Internal Medicine, State University of New York (SUNY) Upstate University Hospital, Syracuse, USA; 2 Rheumatology, State University of New York (SUNY) Upstate University Hospital, Syracuse, USA

**Keywords:** antiphospholipid antibody, caps

## Abstract

Antiphospholipid antibody syndrome (APS) is a multisystem disorder characterized by thromboembolic events in the presence of antiphospholipid antibodies (APLA). Catastrophic antiphospholipid antibody syndrome (CAPS) is an uncommon variant of APS which is associated with widespread coagulopathy that predominantly affects small vessels. Despite maximal treatment, CAPS has a very high mortality rate. We present a case of a 42-year-old woman with a history of APS who presented to our hospital with complaints of epistaxis, hemoptysis, menorrhagia, and shortness of breath. She was diagnosed with CAPS and developed multiorgan failure and sepsis. Despite maximal treatment with immune modulators, she unfortunately succumbed. With this case, we highlight the importance of early recognition of CAPS and review various treatment modalities that have been proven beneficial. Despite these modalities, CAPS remains a therapeutic challenge in many cases and has a high mortality rate.

## Introduction

Antiphospholipid antibody syndrome (APS) is a multisystem disorder characterized by thromboembolic events during pregnancy in the presence of antiphospholipid antibodies (APLA) [1]. Catastrophic antiphospholipid antibody syndrome (CAPS) is an uncommon variant of APS with a prevalence of less than 1% in all patients with APS [2]. It is associated with widespread coagulopathy that predominantly affects small vessels [3]. The diagnosis of CAPS includes evidence of involvement of three or more organ systems and/or tissues, manifesting simultaneously within a week, confirmation by histopathology of small vessel occlusion in at least one organ, lab confirmation of the presence of APLA such as lupus anticoagulant, anti-cardiolipin antibody and/or anti-beta-2-glycoprotein-1-antibody [4]. The majority of patients who develop CAPS have primary APS or systemic lupus erythematosus (SLE). However, a minority of cases are associated with other rheumatological conditions such as Sjogren’s syndrome, rheumatoid arthritis, ulcerative colitis, systemic sclerosis, or relapsing polychondritis [5]. Triggers have been identified in approximately 65% of cases, with the most common trigger being infection [6]. Other triggers include surgical procedures or trauma, withdrawal of anticoagulation medication, SLE flares, and oral contraceptive medications [6]. Pathogenesis of CAPS involves APLA triggering complement activation (C5b-9 deposition) which drives thrombosis of large and small vessels in addition to the release of cytokines which can cause a “cytokine storm” which is responsible for dramatic systemic inflammatory response [4]. The most commonly involved organs with CAPS are kidneys followed by the lungs with renal failure evidenced in approximately 77% of the cases [6]. Despite maximal treatment, CAPS has a mortality of 37% [6]. Many treatments have been tried in the management of CAPS including anticoagulation, steroids, cyclophosphamide, plasma exchange, rituximab, and eculizumab often in combination with varying degrees of success making this an extremity fragile condition [6]. Here, we present a case of a young woman who was diagnosed with CAPS and unfortunately succumbed despite maximal medical management.

## Case presentation

A 42-year-old female with a past medical history of SLE on hydroxychloroquine 200 mg daily and prednisone 10 mg daily for six months, APS on warfarin, valvular heart disease with myxomatous aortic valve with bioprosthetic aortic valve repair, hypertension, hyperlipidemia, cerebrovascular accident and stage II chronic kidney disease presented to the hospital with epistaxis, hemoptysis, menorrhagia, worsening shortness of breath, and weakness along with nausea and vomiting for three days. She was diagnosed with APS seven years prior to admission with triple-positive APLA and lupus anticoagulant positive. 

On day 1, after switching warfarin to enoxaparin for an elective cardiac catheterization, she developed epistaxis and hemoptysis. The patient was initially hemodynamically stable, and her relevant labs from admission are displayed in Table 1.

**Table 1:** Table showing the patient's initial labs on presentation to the hospital INR: international normalized ratio; Anti-Ds-DNA: anti-double-stranded deoxyribonucleic acid.

S. No.	Labs on admission (units)	Value	Reference range
1.	White cell count (10*3/uL)	11.2	4-10
2.	Hemoglobin (g/dL)	8.6	12-16
3.	AST (Aspartate aminotransferase) (U/L)	35	<32
4.	ALT (Alanine transferase) (U/L)	33	<33
5.	Total bilirubin (mg/dL)	1.6	<1.2
6.	Direct bilirubin (mg/dL)	0.4	<0.3
7.	Platelets (cells x10^3^/mm^3^)	52,000	150-400
8.	Creatinine (mg/dL)	2.12	0.7-1.2
9.	INR	4.91	-
10.	Haptoglobin (mg/dL)	<15	30-200
11.	Lactate dehydrogenase (U/L)	547	122-214
12.	Cardiolipin antibody IgA (U/mL)	30	0-11
13.	Cardiolipin antibody IgG (U/mL)	>450	<20
14.	Cardiolipin antibody IgM (U/mL)	35	<20
15.	Beta-2-glycoprotein IgG (U/mL)	>3400	<20
16.	Beta-2-glycoprotein IgM (U/mL)	57.5	<20
17.	Beta-2-glycoprotein IgA (U/mL)	50	0-25
18.	Hexagonal phase phospholipid neutralization assay (sec)	40.3	<8
19.	Rheumatoid factor (IU/mL)	<10	<14
20.	Anti-neutrophilic cytoplasmic antibodies	Negative	Negative
21.	ADAMTS13 (%)	47	>66.8
22.	Anti-nuclear antibody (ANA)	<50	0-49
23.	Anti-Ds-DNA	71	0-99
24.	C3 (mg/dL)	31	90-180
25.	C4 (mg/dL)	3	10-40

Her bleeding was attributed supratherapeutic international normalized ratio (INR) and so the anticoagulation medications were discontinued and she was given 10 mg PO vitamin K for reversal of warfarin. The hemolytic panel revealed low haptoglobin, elevated lactate dehydrogenase (LDH), indirect hyperbilirubinemia, and negative coombs test. A preliminary diagnosis of CAPS was made due to positive APS serology, multiorgan failure, and development of manifestations in less than a week.

On day 3, she was started on intravenous methylprednisolone 60 mg daily and hydroxychloroquine was continued. Atovaquone was started for Pneumocystis pneumonia (PCP) prophylaxis. On day 7, she was transferred to the intensive care unit due to renal failure and the need for dialysis and plasma exchange. A renal biopsy was done to discern the etiology behind rapidly deteriorating renal function. It was reported as early membranoproliferative glomerulonephritis (MPGN)-like picture with positive staining for C3, C4, immunoglobulin A (IgA), negative for complement component 1Q (C1q) with interstitial fibrosis, and intact integrity of the tubule-capillary architecture. It was consistent with features of chronic APS nephropathy, probable class II lupus nephritis, and moderate interstitial fibrosis with tubular atrophy. She was continued on plasmapheresis every 48 hours for a total of 14 days (starting on day 7). Due to a lack of improvement after plasmapheresis, concomitant treatment with rituximab was started on day 12 and she received a total of four infusions of rituximab over the course of the next two weeks, however, due to low cluster of differentiation (CD)19 and CD20 values, further rituximab was not indicated. On day 16, the patient endorsed right upper extremity pain and was found to have bilateral superficial venous thrombosis and heparin drip was initiated.

During her third week of hospitalization, her thrombocytopenia and APLA levels worsened on the days she did not receive plasma exchange (every 48 hours) which suggested that it was a resistant disease despite aggressive measures, and hence the patient was administered eculizumab 600 mg. She was on prophylactic ceftriaxone 1g/24 hours when on eculizumab therapy which was switched to piperacillin-tazobactam when urinalysis (sent prior to eculizumab therapy) came back positive for pyuria. Urine culture was positive for Candida dubliniensis and blood cultures remained sterile. On day 25, the patient started getting hypotensive and lethargic and was found to have lactic acidosis, however, she was clinically worsening with massive volume overload and altered mentation. The patient had initially received three sessions of alternate day hemodialysis after which her renal function improved with improving urine output, however, it did not sustain. Aggressive diuresis and albumin infusions were tried for volume overload without improvement. The deterioration was thought to be due to pre-renal acute kidney injury due to decreased intravascular volume secondary to third spacing in the setting of CAPS and cardiorenal syndrome secondary to her defective aortic valve. The patient was initiated on continuous venovenous hemodialysis. The patient continued to by hypotensive requiring pressor support with norepinephrine (40 mcg/min) and epinephrine (2 mcg/min). Sodium bicarbonate drip was initiated due to worsening acidosis and antibiotics were escalated to meropenem to cover extended-spectrum beta-lactamases (ESBL) and nosocomial gram negatives with the addition of micafungin due to prolonged hospitalization, immunocompromised status, and multiple indwelling lines. The patient was not able to tolerate plasmapheresis due to hemodynamic instability.

By the fifth week, the patient’s condition then continued to deteriorate with lethargy, right gaze preference, bilateral plantar extensor response. She also started having increased work of breathing. Given worsening respiratory and mental status, a possible need for intubation was discussed with the patient’s health care proxy who voiced that the patient would have wished to be a 'do not resuscitate and intubate' (DNR-DNI). The patient eventually passed away. The patient’s family declined autopsy after discussion.

## Discussion

CAPS, also known as Asherson syndrome, represents less than 1% of all patients having APS [5]. Treatment of CAPS is directed towards suppressing cytokine cascade and thrombotic events.

Anticoagulation is directed to inhibit thrombin initiation and promoting clot fibrinolysis. Heparin seems to have an additional advantage as it causes inhibition of APLA binding to target and preventing complement activation [4]. Glucocorticoids and plasmapheresis have shown to improve survival as per observational studies [7-9]. Asherson et al. in a case series of 80 patients noted a recovery in 64% cases who were treated with anticoagulation plus steroids [7]. Seventy percent recovery was noted in 20 patients who were treated with steroids, anticoagulation, and plasma exchange by Asherson et al. [8]. Bucciarelli et al., in their study of 250 patients, found the highest rate of recovery of 78% was achieved when anticoagulation, plasma exchange, and glucocorticoids were used together [9]. Plasma exchange appears to be effective in the management of CAPS due to the immediate removal of APLA from circulation [10]. Intravenous immunoglobulin (IVIG) has also been used in the treatment of CAPS. Its beneficial effects are likely through the direct effects of fragment crystallizable (Fc) receptor blocking antibodies and increasing its clearance. IVIG is also effective in inhibiting CD8 and inhibiting complement system activation [4]. Our case was promptly started on glucocorticoid and plasmapheresis but was found to be resistant to these modalities.

CAPS resistant to standard therapy can be treated with rituximab or eculizumab. Rituximab is a monoclonal antibody against CD20 on B cells and may play a role in treatment for resistant CAPS. A descriptive analysis and review of 20 patients within the CAPS registry showed that there may be some benefit of rituximab in the management of CAPS [11]. However, this study was limited with a small sample size to complete a safety analysis and effectiveness. A pilot study of rituximab found it may be effective in managing only some manifestations of APS [12]. After the initial improvement in symptoms with rituximab, our patient started to worsen and so a decision was made to administer eculizumab to our patient.

Eculizumab is a monoclonal antibody that inhibits the breakdown of C5 to C5a and C5b thus inhibiting the terminal complement system. It is currently used in the treatment of paroxysmal nocturnal hemoglobinuria (PNH), atypical uremic syndrome (aHUS), generalized myasthenia gravis (gMG), and neuromyelitis optica spectrum disorder (NMOSD) [13]. There have been case reports showing the success of eculizumab in the treatment of refractory CAPS [14-19]. Our patient was started on an induction dose of 600 mg with undetectable complement levels which suggested appropriate medication activity. However, our patient developed septic shock from an unknown organism after the first dose of eculizumab despite broad antibiotic coverage, prohibiting her from getting further treatment.

## Conclusions

CAPS is a rare but life-threatening condition and less is known about the disease process. Prompt initiation of anticoagulation, corticosteroids, and plasma exchange in variable combinations remain the mainstay for management, however, the introduction of immunomodulators including rituximab and eculizumab should be considered if conventional management fails. Despite these modalities, CAPS remains a therapeutic challenge in many cases. Current evidence of treatments with immunomodulators are based on case reports and investigation is needed to better understand the role and timing of starting immunomodulators.
